# Feasibility of Proton Beam Therapy for Para-Aortic Lymph Node Recurrence in Patients With Gynecologic Cancer After Pelvic Irradiation: A Case Series

**DOI:** 10.7759/cureus.79514

**Published:** 2025-02-23

**Authors:** Yuka Mizuno, Ayumi Shikama, Kaoru Fujieda, Hiroya Itagaki, Yuri Tenjimbayashi, Yusuke Kobayashi, Tsukasa Saida, Takashi Saito, Hideyuki Sakurai, Toyomi Satoh

**Affiliations:** 1 Department of Obstetrics and Gynecology, Institute of Medicine, University of Tsukuba, Tsukuba, JPN; 2 Department of Radiology, Institute of Medicine, University of Tsukuba, Tsukuba, JPN; 3 Department of Radiation Oncology, Institute of Medicine, University of Tsukuba, Tsukuba, JPN

**Keywords:** gynecologic cancer, history of pelvic irradiation, overlapping irradiated fields, para-aortic lymph node recurrence, proton beam therapy

## Abstract

Aim: Irradiation of para-aortic lymph node recurrences in patients with prior pelvic irradiation raises concerns about overlapping irradiated fields, which should be minimized to reduce severe adverse events. We report six gynecologic cancer patients treated with proton beam therapy for para-aortic lymph node recurrence after pelvic irradiation.

Methods: Six patients who received proton beam therapy for para-aortic lymph node recurrence after pelvic irradiation between 2010 and 2022 were included. Overlapping fields were assessed using the 50% dose distance between the initial and proton therapy fields.

Results: A 5- to 10-mm overlap was observed in three patients. Grade 2 rectal hemorrhage occurred in three patients, grade 3 hematuria in two, and grade 4 sigmoid perforation in one. These adverse events were linked to pelvic irradiation and peritoneal dissemination. Among five patients who died, the median survival after proton therapy was 13 months (range: 9-34). One patient survived with disease at 34 months. Re-enlargement of treated para-aortic lymph nodes was not observed in four patients.

Conclusions: Proton beam therapy was well-tolerated and achieved favorable local control in para-aortic lymph node recurrence after pelvic irradiation. It may be a useful option for gynecologic cancer patients with overlapping irradiated fields.

## Introduction

Patients with gynecologic cancer often develop para-aortic lymph node (PALN) recurrence after initial treatment, posing a significant challenge in the management of gynecologic cancers and impacting the outcomes and treatment strategies of patients. The incidence of PALN recurrence has been reported as 2.1% in patients with cervical cancer [1] and 1-3% in patients with endometrial cancer [2,3]. Although radiotherapy can offer curative treatment for PALN recurrence, irradiation of the PALNs may be avoided in patients with prior pelvic irradiation because the lower line of irradiation for PALN recurrence may overlap the upper line of pelvic irradiation. Attention should focus on the dose to overlapping irradiated fields to mitigate severe adverse events [4].

Proton beam therapy (PBT) delivers higher doses to tumors while minimizing irradiation to surrounding organs. In recent years, the efficacy of PBT for various cancers has been reported [5,6]. However, data regarding the feasibility of PBT for recurrent gynecologic cancers remain limited [7].

This study reports on six gynecologic cancer patients treated with PBT for PALN recurrence after pelvic irradiation to reduce the risk of adverse events.

## Materials and methods

Patients

This study included six gynecologic cancer patients who underwent initial treatment, including pelvic irradiation, at the University of Tsukuba Hospital and were treated with PBT for PALN recurrence between January 2010 and December 2022. All patients were diagnosed with recurrence as lymph node enlargement greater than 10 mm in short diameter on computed tomography (CT) or positive fluorodeoxyglucose uptake on positron emission tomography (PET)-CT before treatment. Clinical information was collected retrospectively from medical records to investigate treatment history and adverse events. The patients with oligometastasis who could be treated as curative irradiation were included. We excluded the patients with an Eastern Cooperative Oncology Group (ECOG) Performance Status (PS) >2, ileus, or severe radiation-related complications.

PBT

PBT planning was performed using CT scans with a slice interval of 2.5-5 mm in the supine position. Captured CT images were transferred to the VQA treatment planning system (Version 1.7 or 2.0, Hitachi, Tokyo, Japan). A passive scattering method was employed, and the proton beam energy was selected within the range of 155-230 MeV. In all cases, a single dose of 2 Gy relative biological effectiveness (RBE) was administered. The initial clinical target volume (CTV1) was defined as the gross tumor volume (GTV) plus a 5-mm margin and the PALN region. While the craniocaudal range of CTV1 was generally established from the L1 to L5 vertebral levels, adjustments were made to the lower boundary of the PBT field to avoid overlap with previously irradiated areas. CTV1 received a dose of 50 Gy (RBE) through two opposing anterior-posterior fields. Subsequently, the clinical target volume for boost irradiation (CTV2) was defined as GTV plus 5 mm, with an additional dose of 6-10 Gy (RBE). Dose constraints for organs at risk were assessed by overlaying the dose distribution from previous pelvic X-ray irradiation with the current PBT distribution. The cumulative dose from X-rays and PBT in overlapping slices was carefully managed. Specifically, the maximum combined dose for the intestines and cauda equina in overlapping slices was limited to 60 Gy (RBE). In slices without overlap, the maximum dose to the intestines was maintained at ≤54 Gy (RBE).

Evaluation

Overlapping irradiated fields were assessed using the 50% isodose line distance between the initial irradiated field and the PBT field. All patients were scheduled for weekly consultations during PBT treatment and follow-up visits every 1-3 months after PBT completion. Acute and late toxicities were assessed using the National Cancer Institute Common Terminology Criteria for Adverse Events (CTCAE), Version 4.0 [8]. Treatment response was evaluated according to the Response Evaluation Criteria in Solid Tumors (RECIST), Version 1.1.7 [9].

Ethics

All procedures were conducted in accordance with the Declaration of Helsinki. This retrospective study was approved by the Institutional Review Board of the University of Tsukuba Hospital (approval number: H24-108). Informed consent for treatment was obtained from all patients.

## Results

**Figure 1:**
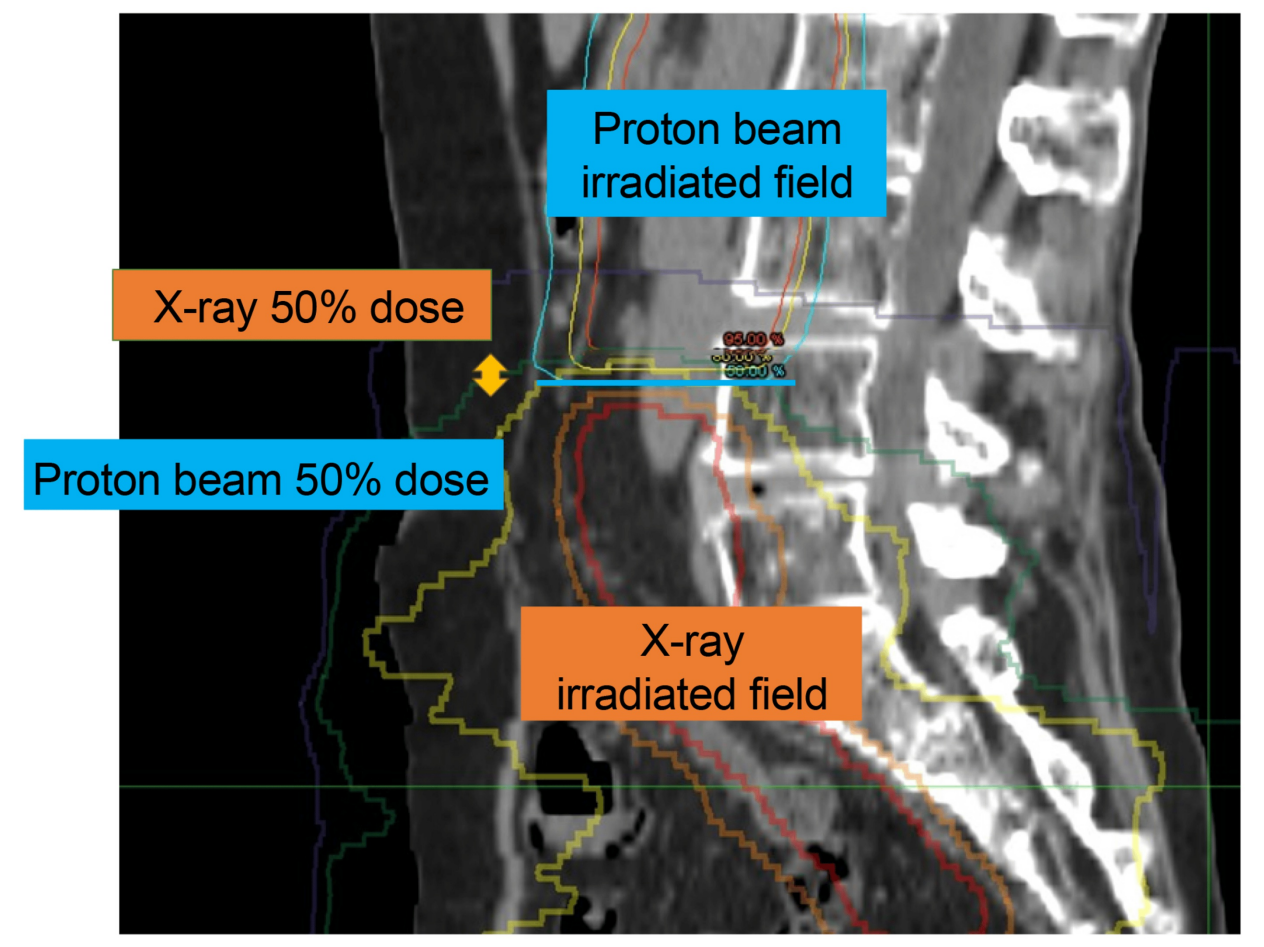
Distance of overlapping irradiated fields Overlapping irradiated fields were assessed as the 50% dose distance between the initial irradiated field and proton beam therapy field.

The patients' background characteristics and treatments are summarized in Table 1. Of the six patients, four had cervical cancer, one had vulvar cancer, and one had endometrial cancer. Initial treatments included concurrent chemoradiotherapy (CCRT) in five patients and postoperative adjuvant radiotherapy in three patients. The upper pelvic irradiated levels were set between L4 and S1, with irradiation doses ranging from 56 to 60 Gy. The median time from initial treatment to recurrence was 18 months (range: 4-24 months) (Figure 1). Among the six patients, three patients displayed overlapping irradiated fields measuring 5-10 mm, which was evaluated using the 50% isodose line distance. The PBT doses ranged from 50 to 60 Gy (RBE). Three patients received CCRT with weekly cisplatin (Table 1; cases 2, 3, and 6).

**Table 1:** Summary of patient background characteristics and treatment CCRT: concurrent chemoradiotherapy; CT: chemotherapy; LN: lymph node; PBT: proton beam therapy; PALN: para-aortic lymph node; IMRT: intensity-modulated radiation therapy ^*1^In case 2, both PALN and mediastinal LN were treated with PBT. ^*2^Endometrioid carcinoma, grade 1, and clear cell carcinoma.

Case	Primary	Histology	Initial treatment			Treatment for recurrence							
			Treatment	Irradiation range	Irradiation dose (Gy/fr)	Age at recurrence (years)	Time from treatment completion to recurrence (months)	Site of recurrence	Short axis of PALN (mm)	50% dose distance between irradiated and PBT fields (mm)	PBT dose (Gy/fr)	Concurrent	Best response
												CDDP	
1	Cervix	SCC	CCRT (X-ray)	Pelvic to L5	50/25	63	23	PALN	10	-2: Separate	56/28	No	CR
2	Cervix	Adenosquamous carcinoma	Surgery → CCRT (IMRT)	Pelvic to L4	50.4/28	59	11	PALN, mediastinal LN^*1^	12	5: Overlap	60/30	Yes	PD (new lesions: supraclavicular LN)
3	Cervix	Mucinous carcinoma (gastric type)	Surgery → CCRT (X-ray)	Pelvic to S1	50/25	63	24	PALN	17	9: Overlap	56/28	Yes	CR
4	Cervix	Mucinous carcinoma (gastric type)	CCRT (X-ray)	Pelvic to L5	50/25	60	4	PALN	15	0: Separate	60/30	No	SD
5	Vulva	Pagetoid spreading tumor	CCRT (IMRT)	Pelvic to L5, inguinal LNs, vulva	60/30	42	14	PALN	8	10: Overlap	56/28	No	PD (new lesions: external iliac LN)
6	Endometrium	Mixed carcinoma^*2^	Surgery → CT → RT (X-ray)	Pelvic to S1	50/25	46	6	PALN	9	Impossible to measure	56/28	Yes	PD (new lesions: lung)
				PALN									

The adverse events after PBT are shown in Table 2. Sigmoid perforation (grade 4) occurred in one patient who developed multiple peritoneal dissemination and massive ascites. The perforation was located outside the PBT field, as confirmed by CT. Two months prior to perforation, the patient underwent a colonoscopy, which revealed radiation-induced proctitis confined to the rectum. Thus, the sigmoid perforation was likely associated with dissemination rather than PBT. Rectal hemorrhage (grade 2) occurred in three patients, and hematuria (grade 3) occurred in two patients. Rectal hemorrhage was diagnosed as radiation-induced proctitis on colonoscopy and hematuria as radiation-induced cystitis on cystoscopy. Both events were attributed to the initial pelvic irradiation since the affected organs were outside the PBT field.

**Table 2:** Summary of adverse events after the induction of PBT PBT: proton beam therapy; N/A: not applicable *Sigmoid colon perforation.

Case	During PBT			After PBT (months)			
	Nausea	Diarrhea	Neutropenia	Rectal hemorrhage	Hematuria	Colonic perforation	Anemia
1	N/A	N/A	N/A	Grade 2 (4)	Grade 3 (1)	Grade 4*	N/A
2	Grade 1	N/A	N/A	N/A	N/A	N/A	N/A
3	Grade 2	Grade 1	N/A	N/A	N/A	N/A	N/A
4	N/A	Grade 2	N/A	Grade 2 (4)	Grade 3 (3)	N/A	Grade 3 (4)
5	N/A	N/A	Grade 2	Grade 2 (4)	N/A	N/A	Grade 3 (4)
6	N/A	N/A	N/A	N/A	N/A	N/A	N/A

The prognosis of patients after PBT is shown in Table 3. The median overall survival after PBT for all six patients was 18 months (range: 9-34 months). Although all patients experienced disease progression after PBT for PALN recurrence, re-enlargement of the PALNs treated with PBT was not observed in four patients, which indicates effective local control of PALN recurrence following PBT.

**Table 3:** Summary of prognosis after PBT PBT: proton beam therapy; LN: lymph node; PALN: para-aortic lymph node; TC: paclitaxel+carboplatin; Bev: bevacizumab; N/A: not applicable

Case	Site of progression after PBT (time in months)	Post-treatment	Time from completion of PBT to post-treatment (months)	Re-enlargement of PALN (months)	Overall survival after PBT (months)	Prognosis
1	Supraclavicular LN	N/A	N/A	No	12	Death
2	Supraclavicular LN	PBT (60 Gy/30 fr)	2	No	34	Alive with disease
3	Lung	TC-Bev	21	No	34	Death
4	Pelvic LN	N/A	N/A	Yes (7)	13	Death
5	External iliac LN	TC	6	No	22	Death
6	Lung	N/A	N/A	Yes (6)	9	Death

## Discussion

This is the first study to investigate the feasibility of PBT for PALN recurrence in patients with gynecological cancers after pelvic irradiation. It offers critical insights into advancing treatment options for this challenging clinical scenario. Re-irradiation was traditionally considered challenging due to the increased risk of radiation-associated complications [4]. However, recent advances in radiotherapeutic techniques, including PBT, have expanded treatment options for recurrence at previously irradiated sites [7].

When irradiation of a PALN recurrence after pelvic irradiation is considered, the lower line of irradiation to the PALN recurrence may overlap the upper line of pelvic irradiation, necessitating careful dose management in the overlapping field. In this study, PBT was chosen for PALN recurrence to avoid adverse events and ensure treatment completion. The unique characteristics of PBT enable a rapid dose escalation at a specific depth while sparing normal tissues [10]. Specifically, the irradiation dose decreases immediately upon entering the body, increases rapidly at the target depth, and ceases thereafter. This allows for increased dose concentration of the tumor and reduced exposure to normal organs. The dose-reduction benefits of PBT are well-documented, with reductions of approximately 32% for the small bowel and 56% for the colon compared to intensity-modulated radiotherapy (IMRT) with X-rays [11]. While PBT to a PALN minimizes unnecessary irradiation to surrounding organs, particularly the intestinal tract and bone marrow, it offers superior sparing of normal tissues compared to X-ray-based techniques. The APROVE (Adjuvant PROton therapy for cerVical and Endometrial cancer) trial, a prospective, single-center, single-arm, phase II trial, assessed the feasibility of postoperative adjuvant pelvic irradiation with PBT in patients with cervical and endometrial cancer, enrolling 25 patients [12]. The results of the study, with a median follow-up of 25.1 months, demonstrated that no patients developed gastrointestinal or urological toxicity of grade 3 or higher, indicating that the treatment was well-tolerated. In the present study, sigmoid perforation was considered associated with peritoneal dissemination since no abnormalities in the sigmoid were observed on colonoscopy immediately prior to perforation. Additionally, all patients who developed rectal hemorrhage and hematuria exhibited findings of radiation-induced proctitis on colonoscopy and radiation-induced cystitis on cystoscopy. These findings were localized within the region of X-ray therapy and outside the PBT field, suggesting that adverse events were less likely to be associated with PBT.

Pelvic irradiation is reportedly more likely to cause myelosuppression, as approximately 60% of adult hematopoietic stem cells are located within the lumbar spine and pelvis [13]. Moreover, extensive radiotherapy has been identified as a risk factor for myelosuppression [14]. In this study, two patients underwent chemotherapy after PBT, and no grade 4 hematologic toxicities were observed, indicating that PBT is unlikely to adversely affect subsequent treatments.

In this study, the local control rate was 66.7% (4/6), and the rate of severe complications (grade 3 or higher) was 33.3% (2/6). Russell et al. reported on re-irradiation using external beam radiotherapy and/or brachytherapy for patients with recurrent cervical cancer who developed recurrence within a previously irradiated pelvic lesion [15]. Among 18 patients treated with external beam radiotherapy and/or brachytherapy, the median dose of whole-pelvis external beam radiotherapy as initial treatment was 43 Gy (range: 24-54 Gy). For patients receiving external beam alone (n=7), the median dose was 62 Gy (range: 39-72 Gy), while for those treated with external beam plus brachytherapy (n=9), the median dose was 40.6 Gy (range: 9-55.2 Gy). Excluding two patients treated with brachytherapy alone, the local control rate was 50% (8/16), with follow-up ranging from 10 to 45 months. However, complications were reported in seven patients (43%), including chronic radiation proctitis (n=2), chronic radiation cystitis (n=1), aseptic necrosis of the femoral neck (n=2), small bowel obstruction requiring surgery (n=1), and rectovaginal fistula (n=1). The results from Russell et al. [15] suggest that using X-rays to deliver sufficient therapeutic doses increases the risk of adverse events. These findings highlight the need for novel radiotherapy modalities capable of delivering high-dose, targeted treatment to disease sites while minimizing adverse events. A study using stereotactic body radiotherapy (SBRT) for recurrent cervical cancer in patients previously irradiated to the pelvis reported a local control rate of 80% (68/85). Chronic toxicity occurring six weeks or more after SBRT was observed in 14 patients (16%) [16]. Additionally, a small study involving 20 patients treated with heavy ion beams, a type of particle beam therapy, reported a three-year local control rate of 61.4%, with no grade 3 or higher adverse events [17]. In the present study, all patients completed PBT as planned, and four of the six patients showed no re-enlargement of PALN. In the two patients who experienced PALN re-enlargement after PBT, the time from completion of initial treatment to recurrence was shorter (four and six months) compared to the other patients (median: 19 months; range: 11-24 months). In a study of re-irradiation using X-rays reported by Russell et al., the median interval from initial treatment to re-irradiation was 14 years (range: 1-37 years) [15]. This suggests that the interval between initial radiotherapy and recurrence may influence radiotherapy sensitivity. If sufficient time elapses following the initial treatment, PBT appears capable of delivering adequate therapeutic doses without significantly increasing the risk of adverse events. However, the effect may be due not only to PBT but also to other irradiation treatments.

This study has some limitations. First, its retrospective nature may have introduced selection bias. Second, data were collected from a single institution, and the sample size was small. Third, the follow-up duration may have been insufficient to comprehensively evaluate safety and effectiveness, as five of the six patients had already died. Further studies with larger cohorts and longer follow-up periods are necessary to confirm the feasibility of PBT for re-irradiation. Despite these limitations, however, this study provides valuable evidence supporting the feasibility of PBT for PALN recurrence.

## Conclusions

PBT is a feasible treatment option for patients with PALN recurrence after pelvic irradiation. The interval between the completion of initial radiotherapy and recurrence appears to influence radiotherapy sensitivity. Thus, when sufficient time elapses after initial treatment, PBT may offer an effective and safe therapeutic approach for gynecologic cancer patients with suspected overlap of irradiated fields. Future studies with larger, multicenter cohorts and longer follow-up periods are essential to validate these findings and establish evidence-based guidelines for the use of PBT in re-irradiation settings. Future research should explore the long-term outcomes and comparative efficacy of PBT versus other advanced radiotherapy techniques.
